# The socialization of hallucinations: Cultural priors, social interactions, and contextual factors in the use of psychedelics

**DOI:** 10.1177/13634615211036388

**Published:** 2021-08-12

**Authors:** David Dupuis

**Affiliations:** Durham University

**Keywords:** Amazon, ayahuasca, hallucinations, hallucinogens, psychedelics, ritual, socialization

## Abstract

The effects of so-called “psychedelic” or “hallucinogenic” substances are known for their strong conditionality on context. While the so-called culturalist approach to the study of hallucinations has won the favor of anthropologists, the vectors by which the features of visual and auditory imagery are structured by social context have been so far little explored. Using ethnographic data collected in a shamanic center of the Peruvian Amazon and an anthropological approach dialoguing with phenomenology and recent models of social cognition of Bayesian inspiration, I aim to shed light on the nature of these dynamics through an approach I call the “socialization of hallucinations.” Distinguishing two levels of socialization of hallucinations, I argue that cultural background and social interactions organize the relationship not only to the hallucinogenic experience, but also to its very phenomenological content. I account for the underpinnings of the socialization of hallucinations proposing such candidate factors as the education of attention, the categorization of perceptions, and the shaping of emotions and expectations. Considering psychedelic experiences in the light of their noetic properties and cognitive penetrability debates, I show that they are powerful vectors of cultural transmission. I question the ethical stakes of this claim, at a time when the use of psychedelics is becoming increasingly popular in the global North. I finally emphasize the importance of better understanding the extrapharmacological factors of the psychedelic experience and its subjective implications, and sketch out the basis for an interdisciplinary methodology in order to do so.

## Introduction

Identifying the underpinnings of the effects of so-called “psychedelic” or “hallucinogenic” substances under experimental conditions has historically been deemed a great challenge, as these substances are known for their strong conditionality on cultural context (Langlitz, 2012). While Timothy Leary (1961) coined the terms “set” and “setting” for these extra-pharmacological factors shaping the drug experience, Claude Lévi-Strauss proposed to consider hallucinogens as “triggers and amplifiers of a latent discourse that each culture holds in reserve and for which drugs can allow or facilitate the elaboration” (Lévi-Strauss, 1970, p. 13).

Comparative studies of the uses of hallucinogens (Dobkin de Rios, 1984; Furst, 1976) show that some features of the experiences induced by these substances are similar across cultures (e.g., entoptic—i.e., visual hallucinations composed of geometric patterns), while others vary extensively cross-culturally (e.g., subjective feeling tone, meaning, or content of the hallucinations). Many ethnographers have observed homogeneity in the features of the hallucinatory experience within the same culture, which has led them to defend a culturalist approach to psychedelic hallucinations (Brown, 1978; Dobkin de Rios, 1972; Langdon, 1979; Lévi-Strauss, 1970; Mooney, 1896; Reichel-Dolmatoff, 1972; Wallace, 1959). For instance, terms such as “culturally influenced visions” (Langdon, 1979) or “stereotypic visions” (Dobkin de Rios, 1974) have been used to emphasize the role of cultural variables in the psychedelic experience. Although some candidates have been proposed to shed light on the factors of this enculturation of the hallucinogenic experience, such as mythological and cosmological knowledge (Reichel-Dolmatoff, 1972), kinship system, iconographic representations (Langdon, 1979), ritual interactions, and verbal exchanges (Dupuis, 2019), the vectors by which the features of hallucinations are structured by social factors have been so far little explored and require further study. Using data collected during an ethnographic study conducted in the Upper Peruvian Amazon over the past decade, I will draw some leads in order to shed light on the underpinnings of these dynamics, which I propose to refer to as the “socialization of hallucinations.”

Comparative ethnographic observations I have carried out over the past decade in various institutions offering ritual uses of the psychedelic brew ayahuasca^1^ in the Upper Peruvian Amazon suggest that cultural and symbolic elements (such as cosmological and etiological theories) as well as the characteristics of the ritual devices specific to these institutions strongly influence the formal characteristics of the hallucinations (i.e., visual and auditory imagery locally labelled as “visions” and “voices”) perceived by the participants. These observations suggest that the vectors of socialization of hallucinations are likely to be understood through the consideration of the symbolic background and the social interactions surrounding the use of psychedelics. To illustrate this point, I employ an anthropological approach rooted in interactionism and social constructionism (Becker, 1963) in dialogue with phenomenology and recent models of cognition of Bayesian inspiration (Carhart-Harris et al., 2014; Corlett et al., 2009).

Distinguishing two levels of socialization of hallucinations, I argue that cultural background and social interactions organize not only the relationship to the hallucinogenic experience, but also to its very phenomenological content. I account for the underpinnings of the socialization of hallucinations proposing two candidate factors for each of these two levels of enculturation: the education of attention and the categorization of perceptions for the first one; the shaping of emotions and expectations for the second.

Considering psychedelic experiences in the light of their noetic properties and cognitive penetrability debates, I show that hallucinogenic rituals are powerful vectors of socialization and question the stakes of this claim with regard to religious transmission. I interrogate the ethical stakes of these observations, at a time when the use of ayahuasca and other psychedelic substances is becoming increasingly mainstream, in the context of the emergence of shamanic tourism and the revival of psychedelic science. I emphasize the importance of better understanding the extrapharmacological factors of the psychedelic experience and its subjective implications and sketch out the basis for an interdisciplinary methodology in order to do so.

## Method and materials

To explore the aforementioned points, I will draw on data collected during 18 months of ethnographic fieldwork divided into three research stays, between 2008 and 2013. The research study was conducted in the San Martín region (High-Amazon region of Peru), mainly in Takiwasi, one of the most famous “shamanic centers” of the region. During the first four-month stay in 2009, I focused on the treatment of addictions proposed by Takiwasi. I used different methods of data collection, among which the study of daily and ritual interactions through the observation of participants played a central role, along with interviews and life stories of about 10 drug-dependent patients and the main actors of the institution (ritual specialists and psychologists, etc.). The second six-month stay was in 2011, during which I attended four “seminars” offered to foreign clients. In each of these seminars there were between 15 and 20 participants. I shared the participants’ daily and ritual activities, as well as the word groups. I also conducted individual interviews with around 30 participants, and they shared their life stories with me. The eight-month final visit was in 2013. On this occasion, I attended three more “seminars,” that included a total of 45 participants. I also conducted weekly interviews with two drug-addicted patients who were hospitalized for six months and, more occasionally, with other patients. Since then, I have conducted through interviews a long-term follow-up of some of Takiwasi’s clients through interviews in Europe and in Latin America.

This research study was also complemented with a comparative study, aimed at becoming more acquainted with the specificities of the institution under study in relation to the shamanic practices of the mestizo or Lamista people of the region. To this end, I carried out several short field surveys in the San Martin region (San Roque de Cumbaza, Chazuta etc.), accompanying in their daily and ritual activities mestizo and indigenous healers offering their services to local people within their village or to a national and international clientele within shamanic centers.

This ethnographic research study, which in France does not require the approval of an ethics committee, complied with the principles of “informed consent” and was conducted with voluntary participants who were aware of the study objectives and knew that they could leave the investigation at any time if they so desired.

### Takiwasi: A shamanic center in the Peruvian Amazon

Participation in exotic rituals perceived as traditional and invested as therapeutic, religious, or personal development practices have been increasingly popular with the Western public since the second half of the 20th century. Fueled by the craze for the psychotropic beverage ayahuasca as well as by the mythified image of the so-called primary forest, an influx of travelers has headed towards the Peruvian Amazon from the 1990s onwards (Labate & Cavnar, 2014; Labate & Jungaberle, 2011; Labate et al., 2016).

Many reception centers for this clientele have appeared on the edge of the region’s metropolitan areas. These “shamanic centers” (Losonczy & Cappo, 2013), most often based on the partnership between Westerners and Métis or indigenous locals, offer an international clientele the opportunity to participate in ritual activities inspired more or less freely by the practices of the Peruvian *curanderismo*,^2^ such as the ritualized use of ayahuasca.

Founded in 1992 by French doctor Jacques Mabit as well as by Peruvian and Spanish collaborators, the therapeutic community of Takiwasi is both an addiction treatment clinic and one of the main places in the region hosting Western trato “meet ayahuasca.” The premises are located on the outskirts of the city of Tarapoto, within a two-hectare site.

Since its creation, the institution has developed a therapeutic device characterized by the reappropriation of elements of the indigenous pharmacopoeia, such as emetic plants or ayahuasca. A team of doctors, psychologists, and ritual specialists offers psychological support, medical follow-up, and ritualized practices inspired by the Amazonian tradition, combining ritual and discursive elements from the region’s indigenous and metis shamanism, Catholicism, and New Age spirituality. These services are proposed in three different modalities: addiction treatment, which involves a nine-month internment; outpatient treatment; and “personal development seminars.”

Jacques Mabit is now the main authority in Takiwasi; the French doctor is the last of Takiwasi’s founding members to still hold responsibilities within the institution. Since then, he has been the bearer of ritual authority, and all important decisions are subject to his approval. Apart from the Métis and indigenous curanderos sometimes recruited by the institution, only a few people regularly officiate during the rituals. More recently, a priest has joined the team: he provides spiritual direction services as well as a weekly mass in the Takiwasi chapel, and regularly participates in the institution’s ritual practices.

Bringing together about 15 participants for a two-week period, the seminars offered by Takiwasi include various practices inspired by Peruvian shamanism such as rituals focused on the ingestion of emetic plants and ayahuasca, and a few days’ retreat in the jungle involving the consumption of other vegetable preparations (*dieta*). Participation in these activities, accompanied by introductory lectures, speech groups, and individual interviews, requires compliance with various food restrictions (pork, spicy condiments, alcohol), as well as romantic relationship and sexual prohibitions.

Seminar participants, both men and women, between the ages of 20 and 60, come from the middle and upper classes in the urban areas of French-speaking Europe and Latin America. They most often report a life journey marked by the accumulation and repetition of different registers of misfortune (death, chronic pain or pathology, accidents, academic or professional difficulties, “loss of meaning”), the resolution of which is presented as the main reason for their coming. The resistance of these difficulties to the treatments offered by medicine and the dominant forms of psychotherapy (psychoanalysis, CBT, etc.) has most often initiated a process of experimentation with alternative therapies, of which the stay in the Amazon is a step. Coming to Takiwasi also reflects a form of “New Age” religiosity characteristic of Western modernity based on the accumulation of “spiritual experiences” from various cultural backgrounds as well as on a modular, individual, and irregular practice (Hervieu-Léger, 1999).

### The ayahuasca ritual in Takiwasi

At Takiwasi, the first ayahuasca ritual takes place on Wednesdays, the third day of the seminar, after the group’s participation in a purging ritual and several conferences, during which Jacques Mabit presents the program of the seminar as well as some elements of his etiological theory, including contamination by invisible pathogens of a “spiritual” nature. The experience of ayahuasca is then described as enabling the purification of the participant and their encounter with the “spiritual world” and usually invisible beings. Following an approach inspired by the psychotherapeutic use of hallucinogenic substances (Grof, 1980), the ayahuasca experience is also presented as a psychological catalyst aimed at facilitating insights, emotional catharsis, childhood regression, and understanding of the tendencies and dispositions that organize the daily behavior of the participant.

Shortly after nightfall, participants are invited to join the main *maloca*,^3^ where about 15 cushions are placed in a semi-circle. After immersing themselves in a purifying and protective bath of plants, participants take their seats around the ritual specialists. They have reserved places under icons representing Christ, the Virgin, and Saint Michael, in front of which are placed their ritual tools (*mesa*). The ritual is frequently preceded by a reminder of the rules, such as body position requirements: participants are not allowed to sit, and have to keep their back straight, refrain from lying down, and abstain from sleep in order to “face what arises.” Participants are also invited to refrain from making noise, speaking, singing, or interfering with, touching, or talking to a neighbor. Ritual specialists usually justify these provisions by the fact that subtle pathogenic elements are likely to be transmitted between participants through touch or sound. Participants are encouraged to “formulate an intention” before drinking ayahuasca, but not to focus on it during the ritual. In case of difficulties, participants are allowed to call upon the officiants. Buckets are available if they feel the need to vomit, while forcing vomiting is not recommended. In case of emergency, it is possible to go to the toilets, located outside the *maloca*, but it will be necessary to report it and wait until a “cleansing” (*sopladas* of perfumes or tobacco) has been carried out by an officiant before returning to the ritual space. These rules will be in place until a ritual specialist turns the light on again, a gesture that marks the end of the ritual.

After these few words of the master of ceremony, a ritual specialist grabs a censer in which *palo santo*^4^ burns, goes around the *maloca*, then stops in front of each participant whom he invites into the smoke presented as purifying and protective. Jacques Mabit then makes a few gestures to materialize the limits of the space occupied by the participants, using holy water and salt, which he spreads behind and between them. These actions, presented to the participants as delimiting a “ritual circle” capable of protecting them from negative interactions, therefore imply compliance with the rules concerning their entry and exit. The master of ceremonies then sings several songs (*icaros*) and blows tobacco smoke (*mapacho*)^5^—again presented as protective—on his ritual tools, including the bottle containing ayahuasca. Everyone is then invited, one after the other, to come and have a cup of the drink. The light is then turned off. Several songs arise, recited by the various officiants, while one of them walks by the participants, blowing tobacco smoke on the chest, fontanel, and hands (*soplada*) of each of them. Then comes the recitation of an exorcism prayer in French, other songs, as well as a second series of *sopladas*, this time made with the help of *agua florida*.^6^ The officiants’ songs, recited in Spanish, Quechua, and French, will then follow one another until, six to eight hours later, the effects of the drink dissipate.

### The hallucinogenic experience in Takiwasi

According to the participants’ testimony, the ingestion of ayahuasca most often involves specific physiological reactions such as nausea, vomiting, diarrhea, sensations of temperature variations, dizziness, and heart rhythm changes. Anxiety, confusion in the perception of time and space, altered proprioception, and dissociative disorders are common, while perceptions and emotions are generally exacerbated. Perceptions of visual, tactile (feelings of touching, grazing), olfactory, taste, and auditory (sounds, melodies, voice) hallucinations are also regularly reported. Participants thus testify to the production of a rich mental imagery—a property that has given the plant preparation its reputation as a “hallucinogen.” These mental images, here called “visions,” are generally composed of bright and colorful “geometric” shapes. Visual hallucinations can also take on more figurative forms. Many participants thus report the perception of animals, anthropomorphic beings, or mixed beings combining human, plant, and animal elements that emerge and evolve within rich visionary tableaux.

The formal characteristics of these visionary narratives are relatively consistent with the descriptions collected by psychologists during experimental studies on hallucinations (Siegel, 1977) as well as with comparative work (Shanon, 2002) or ethnographic reports concerning the use of psychotropic beverages composed of banisteriopsis (Chaumeil, 1983; Déléage, 2009; Descola Philippe, 1993; Dobkin de Rios, 1972; Reichel-Dolmatoff, 1978). However, the testimonies of the clients of the seminars offered by Takiwasi show some specific features that distinguish the center from these contexts. As illustrated by the testimonials collected during the survey, seminar participants indeed frequently report the perception of threatening entities referred to as “demons,” which they most often describe in our interviews as fighting against protective entities such as spirits of nature or entities of the Christian pantheon. In the words of ceremony participants:

I have often seen demons. They were floating in the air, next to people, they wanted to get into their bodies or they were already inside. I felt negative presences several times next door watching me and not wanting to let go.

I saw demons eight or nine times in ayahuasca sessions, it started around the second or third month of treatment. Most often they would observe me, hide, and try to intimidate me.

At one point I had a vision of the archangel Saint Michael who was piercing a demon with his sword, as in the religious images. Later I felt the presence of Christ, who looked behind my back, where chains were hung connected to a cage. I saw my demons laughing because I had to drag my cage to move forward in life. They jerked me around all the time, like they were raping me. (…) When Christ saw the chains and the cage, he said it had nothing to do here and kicked to kick it all out.

There were all these demons “parasitizing” [sic] me inside, but I saw the ayahuasca that was chasing them, like a lot of little bright snakes inside my body that were circulating and cleaning all that up. (…) Later I saw ayahuasca. She was a kind of woman with a snake-like lower body, showing me how the demons got in, what I had done, and therefore what I had to do to stop them from entering.

These visionary narratives vividly illustrate the cosmological and etiological theory that characterizes Takiwasi and distinguish this institution from both the Peruvian *curanderismo* and other shamanic centers. The central element of this theory is the concept of “infestation.” Borrowed from Catholic theology, this concept initially refers to a more minor and common mode of demonic influence than possession, characterized by the presence of a demonic entity abusing the subject by affecting their health, faith, or thoughts (Du Clos, 2007). In Takiwasi, infestation is thought of as a parasitic relationship with one or more malicious supernatural beings of a demonic nature. This condition, which is also presented as the cause of physical and psychological disorders, is thought of as the consequence of the transgression of taboos (drug use, sexuality, magic practices, spiritualism, etc.), contact with places or people, or transmission through filiation. Finally, this condition is presented as requiring specific treatment, implying the purification of the patient through the absorption of emetic preparations or ayahuasca, as well as through practices proposed by the Catholic Church, such as exorcism (Dupuis, 2018).

This theory, developed by the main actors of the institution over the past 20 years, reveals the increasing use of a Catholic doctrinal body and the ecclesial institution, which has profoundly influenced the form and function of the practices proposed by Takiwasi (Dupuis, 2017). The ayahuasca ritual is indeed now characterized by the use of exorcism prayer, the crucifix, holy water, and the mobilization of the main figures of the Catholic pantheon. In this sense, Takiwasi offers a very original example of contemporary recompositions of so-called “shamanic” labelled practices centered on the use of ayahuasca in the Amazon (Labate & Cavnar, 2014; Labate & Jungaberle, 2011; Losonczy & Cappo, 2013).

The above testimonies thus appear to be a visionary staging of the cultural and ritual features of the institution. The observation of experienced users (drug-addicted patients at the end of treatment, clients of several successive seminars) as well as the long-term follow-up of participants also underline the progressive dimension of the emergence of these visionary patterns, which invites us to approach it as the result of a learning process.

### Framing the hallucinations: Education of attention and categorization of perceptions

I observed during the course of the investigation that the predominance of demonic visions gradually marks the visionary testimonies of the participants. As illustrated by these testimonials collected during interviews with participants at the end of the seminars, this apparent homogeneity hides important formal variations:

Demons (…) looked like entities that had some kind of animal attributes that were nested within each other. There was an elephant’s trunk with tentacles and stuff like that. Already, it’s very ugly to look at and there’s a feeling, a repulsion. It’s as if it wants to feed on you, as if it wants to penetrate you, or at least it’s in you.

That night I started sweating. I saw a lot of flames, I was hot. Then I saw the devil, a vision that passed like a flash: an old man’s face crumpled on a background of flame. I was terrified, it was really horrible, I knew it was a demon. Thirty minutes later the same vision came back, and this time it was blowing gently on one of my ears. I don’t know why, but it was horrible, I was panicked.

Some of the participants who evoked an encounter with demons concede that they did not see anything strictly speaking:

At one point, the demons came: I was vomiting things, I felt that false lights were leading me to dark things. I couldn’t see anything specific but I was afraid, it gave me terrible anxieties. The demons, they attack you and they scare you because the ayahuasca clears them, they cheat you so that you stop the work that expels them.

It may seem surprising that despite their formal diversity, these hallucinatory experiences are all interpreted as signaling the presence of the same category of supernatural entity: the “demon.” The feeling of familiarity leading to the recognition of the demon’s presence thus seems to be based less on a specific visual content than on the emotions that accompany it, which appear to be very homogeneous. Like the above examples, fear or disgust almost always accompanies the demonic experiences.

To shed light on these recurring patterns, we must focus our attention on the symbolic and interactional contexts that surround the visionary experience, and especially on the discussion groups that follow it. The day after the ayahuasca ritual, the participants are in fact invited to meet in the early afternoon to participate in a discussion group called “post ayahuasca.” In the presence of Dr. Mabit and one of his assistants, everyone is asked to recount in turn and in front of everyone his or her experience of the ayahuasca. The ritual specialists then give some comments to the participant.

Through these comments, ritual specialists frequently invite participants to consider some aspects of their experience as signs of the presence and influence of protective or malicious supernatural entities. Depending on the comments given to the participants, ritual specialists, who position themselves as the holders of a discernment skill initially denied to the participants, gradually transmit the criteria that will allow the latter to identify the somatic, emotional, and cognitive signs manifesting the presence and nature of supernatural entities. These narrative interactions will therefore have a great influence on the participant’s subsequent ritual experience, which tends, as the previous examples illustrate, to be organized according to the script proposed by ritual specialists.

As illustrated by the previous testimonies, participants frequently categorize their hallucinations, perceptions, and mental states in a dualistic manner, attributing them to supernatural entities with antagonistic intentions. Visions accompanied by fear, guilt, or confusion are thus almost always interpreted as a sign of the presence of malicious entities seeking to disturb the participant, while those accompanied by relief, joy, and appeasement will instead be attributed to the action of benevolent and protective entities such as the spirit of ayahuasca, animal spirits, or entities of the Catholic pantheon.

Ayahuasca, made present to the participant by its ingestion and by the actions and speeches of ritual specialists, is very frequently perceived as the origin of the participants’ “visions.” Ritual actions seem to play a leading role in this respect. The actions surrounding the service of the psychotropic beverage as well as the ritual songs, which consist of long addresses to invisible entities, notify the presence of invisible entities, in the first place including “the spirit of ayahuasca,” a benevolent entity aiming at the purification, education, and protection of the participant. The actions elaborating the ritual space as well as the rules related to the crossing of borders that they draw postulate the circulation of invisible agents in and between the built spaces (*maloca*, participants’ bodies), presented in the form of pathogenic contamination. The demonic nature and malicious intentions of these agents will typically be explained by the recitation of exorcism prayer. In this regard, it should be noted that the master of ceremony recites the exorcism prayer at a speed that makes the speech difficult for a human ear to hear: the master therefore positions themselves as addressing not the assembly but invisible beings whose presence is thereby implied.

Beyond this theatrical aspect of the ritual, the interactions between healers and participants also play a key role in the organization of the hallucinogenic experience. I have shown in previous work (Dupuis, 2018) that the ritual specialists’ treatments (exorcism techniques including prayers recited aloud, use of holy water and crucifix, etc.) of participants who transgress the ritual rules by moving, showing agitation, shouting, or speaking invite the patients concerned as well as the entire ritual audience to interpret their experience through the prism of cultural propositions based on the infestation by demonic entities. Thus, without the need to perceive them clearly, participants are invited to assume the presence of invisible beings with whom ritual specialists seem to be in a relationship and with whom they are encouraged to think of themselves as related.

The context surrounding the hallucinogenic experience is highly likely to alter metacognitive processes (Proust & Fortier, 2018). The ritual context induces in particular absorption—being caught up in the inner experience (Lifshitz et al., 2019)—as an increase and orientation of attention. This fosters the identification of certain perceptions or specific mental states, in a process reminiscent of Ingold’s notion of “education of attention” (Ingold, 2001). The learning of discernment criteria and ritual interactions might in turn impact the inferential processes managing the categorization of perceptions and mental states, which are therefore prone to be interpreted as signs of the presence, agency, and nature of culturally postulated supernatural entities.

By this account, the regularities of visionary testimonies appear to be the result of an education of attention and a transformation of the process driving the categorization of perceptions. These two first building blocks of the socialization of hallucinations (i.e., the education of the attention and the modification of the procedures of categorization of perceptions) are made possible by participation in speech and ritual interactions surrounding the hallucinogenic experience. Following the participants’ testimonies, it seems that a feeling of familiarity prompts them to identify some hallucinations as a sign of the presence of culturally postulated supernatural entities. This identification process seems to be less based on the formal characteristics of the visual and auditory imagery than on the articulation of the hallucinogenic experience with the implicit logic of the ritual interactions and the normative criteria disseminated during the conferences and discussion groups. While the ingestion of ayahuasca gives rise to intense and confusing perceptual and emotional arousal, ritual rules (darkness, prohibition to move around or interact with other participants, etc.) lead participants to focus their attention on their inner experience; the ritual and verbal interactions, in turn, invite participants to organize these experiences through the prism of the cultural categories proposed by the social group.

In her seminal work on voice-hearing, the anthropologist Tanya Luhrmann (2012) shows how differences in the practice of prayer produce differences in how God becomes present to those who pray. Among many other experiences, God manifests to believers through auditory-verbal hallucinations (“voices”). More recently, Luhrmann has insisted on the importance of metacognitive processes in prayer practice, and has proposed to think of prayer as a metacognitive act that alters the relationship of those who pray to their own mental domain (Luhrmann, 2018). The redirection of attention and invitation to absorption have been proposed as the main features of this metacognitive act. Comparing samples from USA, India, and Ghana, Luhrmann and colleagues (Luhrmann et al., 2015) also suggest that the voice-hearing experiences of persons who met the inclusion criteria of schizophrenia are shaped by different “cultural invitations,” which they define as cultural variations in ways of thinking about minds, persons, and supernatural beings. Luhrmann and colleagues have argued that cultural invitations seem to strongly influence the relationship to the voice-hearing experience and how it is categorized and interpreted by voice hearer. The authors found that, in California, people seem to be more likely to describe their voices as intrusive unreal thoughts, while in South India, voices are mainly described as providing useful guidance.

My findings are consistent with these observations. The education of attention and invitation to absorption as well as the influence of the local ontological and etiological theories in the categorization of mental events appear to be the main vectors through which cultural background and social interactions shape the subject relationship to the hallucinogenic experience.

Despite these emerging models and findings, more work is needed to further elucidate the relationships between hallucinations and culture (Larøi et al., 2014). While these new paradigms shed light on how hallucinations are detected, categorized, and interpreted, ethnographic observation suggests that the very content of the hallucinogenic experience (not just the relationship with it) may be influenced by the context in which they emerge. It is this second level of socialization of hallucinations that I will now attempt to shed light on.

### Shaping the phenomenological features of hallucinations

#### Sensory stimulation and emotional arousal

At Takiwasi, many participants reported that during ayahuasca rituals, some interactions with ritual specialists radically and almost immediately transformed the content of their psychedelic experience. In particular, people emphasized the impact of ritual songs (*icaros*) and perfumes (*agua florida* and other) as well as tobacco smoke, which, as we have seen, is blown over their bodies several times during the ritual (*sopladas*), on their visual and auditory imagery:

When Jacques recited the song of the Virgin, I felt really bad, I wanted to vomit, I was agitated, I wanted to leave the maloca, it was swarming.

I had disgusting visions, it was swarming. I saw insects swarming and climbing on top of me, I couldn’t get out of it … And then Rosa started to sing, a calm, very sweet song. (…) I felt like it was for me. It really calmed me down and I found myself in a beautiful meadow, clear, I could feel the wind on my face, I was there.

I was really angry, I’ve been locked in there for a while. Fabienne came and blew perfume on me, and I could feel the liquid releasing the anger. I calmed down more and more, while in the visions I saw a volcano erupting and going off.

These brief testimonies recall the importance, frequently reported by ethnologists, of the role of singing (Beyer, 2011; Brabec de Mori, 2009; Chaumeil, 1983, 2010; De Rios & Katz, 1975; Townsley, 1993) and perfumes (Beyer, 2011; Luna, 1986) in the ritual devices surrounding the use of ayahuasca in the mestizo and indigenous shamanisms of the Peruvian Amazon. Anthropologists studying the ritual use of hallucinogens have underlined that perfumes, music, and sound play a driving role in structuring the hallucinogenic experience, allowing, to some extent, for the management of its symbolic content.

These foregoing testimonies also raise questions about how olfactory and auditory, but also tactile, visual, and taste experiences interact with each other. Comparing the phenomenology of ayahuasca experiences in various cultural contexts, Shanon (2002) argues that these cross-modal perceptions (i.e., synesthesia) are some of the most important features of the ayahuasca experience. More importantly for our purpose, these observations raise questions about how sensory stimuli such as songs and perfumes influence visual and auditory imagery. I suggest that this is partly based on the ‘emotional’—that is, physiologically arousing—properties of strong sounds and smells and their hedonic tone (pleasant or unpleasant).

Perfumes, like music, are indeed known for strongly affecting the emotional state of the person who perceives them (Gabrielsson & Lindström, 2001; Herz, 2002; Radford, 1989; Soussignan, 1997). The perception of music is for instance likely to foster the emergence of mental images (Osborne, 1981) whose content can be considered either as indexed to the emotions the music evokes in the listener or as the source of those emotions (Juslin & Sloboda, 2011). The power attributed to *sopladas* by some participants could be understood by the properties of the substances used, all of which are highly odorous (tobacco smoke, *agua florida*). Exposure to a scent perceived as pleasant often results in a sense of well-being and induces emotional, behavioral, and cognitive effects associated with this state (Herz, 2002). Similarly to music (Juslin & Sloboda, 2011), smells also have a well-known relationship with memory: in a direct way, they activate the recollection of scenes where the same smell has been perceived. This recollection most often involves memories with a high emotional content: pleasant smells or music tend to trigger memories associated with an emotion of well-being without any necessary link to the sensory input that activated this memory, and an unpleasant input of memories associated with emotions of unease.

I suggest that it is through these ‘emotional properties’ that perceptual stimuli influence the content of visual and auditory verbal imagery during the psychedelic experience. In the context of the ayahuasca ritual, the emotional properties of fragrances and music meet the synesthetic properties of the psychotropic beverage, which promotes cross-modal perceptions. The psychedelic brew, which generally increases emotional and perceptual sensitivity, also stimulates the production of rich visual and auditory imagery, the content of which is shaped by the emotional state of the subject.

This hypothesis requires further study, in particular on the clinical implications of these remarks in the treatment of hallucinatory disorders in psychosis (Dupuis, 2021). Indeed, the view presented here may help shed light on the ability of ritual specialists to radically modify the content of the hallucinogenic experience through their chanting and *sopladas*. This experience leads the participants to concretely verify the healers’ ability to manage their own hallucinogenic experience, which the latter most often attribute to powers related to their privileged relationships with supernatural entities. This is particularly noteworthy, in that this experience frequently induces a deferential position of the participants towards the ritual specialists’ epistemic authority. By establishing the authority of ritual specialists, this aspect of the psychedelic experience is likely to facilitate the dynamics of the socialization of hallucinations.

#### The shaping of expectations

Apart from these sudden transformations in visual and auditory imagery, my observations of the course of the participants’ experiences underline a progressive homogenization of the formal characteristics of the hallucinogenic experience during ayahuasca rituals, which are organized according to a few recurring patterns. This trend is illustrated in particular by the stereotypical nature of the visions that index the presence of supernatural entities. The perception of demons, entities of the Catholic pantheon, or a protective snake was thus reported by many participants during our interviews:

I saw a snake come down to me and start talking to me. He asked me what I was doing there, what I was looking for. I could talk to him; it was like telepathy.

During the diet, I prayed a lot. At one point, in full prayer, I saw something like a hole in the sky and then I heard a voice that said to me, “Get up, look at me.” There I visualized in my head a man on a golden throne with the Virgin who repeated to me “look at me, in my eyes” (…) Then I had a vision with the archangel Saint Michael who pierced a demon, as in the religious images, (…) he was blond, curly, but I didn’t see his face too much.

These testimonies are striking first and foremost for their congruence with Catholic iconography, which the participants, most often coming from this religious culture, consistently highlighted during our exchanges. These observations point to the influence of the shared culture of the participants, whose visionary scene seems to constitute the projective support. Many of them, when describing their “visions” of demons, do so by referring to Christian and popular iconography related to the cultural pattern of demonic possession (as one participant says, “at one time I saw myself with a devil’s tail and with the face of the girl from the movie *The Exorcist*”).

Finally, “the spirit of ayahuasca” is often seen in the form of an encounter with a snake, or a woman with vegetal and ophidian features. This scene constitutes one of the major tropes of iconography specific to the shared culture of shamanic tourism clients. This iconography, composed of paintings, drawings, computer-aided drawings, and audiovisual productions—documentary or fiction (Bernardi, 2005; Cheyssial, 2003; Kounen, 2004)—is easily accessible on the Internet. These images, strongly influenced by the works of native or metis Peruvian artists such as Pablo Amaringo (Luna & Amaringo, 1999), and psychedelic art patterns often decorate the autobiographical books and online forums of shamanic tourists, as well as the websites of “shamanic centers.”

These observations invite us to approach the recurring patterns of hallucinatory images as an example of an invasion of perception by culture, and thus bring to mind the case of *pareidolias*—that is, the tendency to infer (incorrectly) personalized meaning or patterns in features of the world. Although *pareidolias* are probably universal and are known to reflect innate dispositions (e.g., attentional bias for faces, gaze, or intentions), their content is also frequently shaped by cultural priors and acquired knowledge. Like *pareidolias*, visionary images are often described, as in the previous testimony, as arising from a disturbed, confused, or ambiguous visual stimulus (darkness, perceptual blurring), which is then reduced to an already known form. The case of *pareidolias* testifies to the weight of past experiences, acquired knowledge, expectations, predispositions, beliefs, or emotional states on perception. Perception in this sense cannot be understood as the passive reception of information by the sensory organs. In this perspective, hallucinatory perceptions can be cast as a particular case of Bayesian inference (Knill & Richards, 1996), a predictive bet emerging from the encounter between expectations and sensory stimuli, which are then reduced to forms shaped by innate dispositions (e.g., anthropomorphic hyper-sensing of agency), cultural knowledge, and past experiences. The use of hallucinogenic substances (such as ayahuasca) as well as contexts of social and sensory deprivation (such as the “dieta,” a retreat of a few days in the jungle involving the consumption of other plant preparations involving relational, dietary, and sexual prohibitions) seems to favor, like in psychotic states, a disruption of the relationships between predictions, expectations, and the perceptual environment, leading to the emergence of perceptions that are deeply influenced by culture, which are commonly called “hallucinations”(Corlett et al., 2009).

The social device surrounding the use of ayahuasca therefore appears not only to be capable of inducing hallucinations and organizing the relationship maintained with them, but also to gradually homogenize the very content of the hallucinatory experience. The duration of exposure to the proposed practices seems to be a driving force in this regard. The degree of stereotypization of hallucinatory patterns seems to be much higher among experienced users than among newcomers. This observation suggests that the mechanisms that ensure the association of ambiguous visual and auditory imagery with an identifiable element are highly plastic and likely to be gradually modified and patterned by the social environment. The discursive and ritual interactions—as well as the iconographic elements—that frame the hallucinogenic experience here, in that they shape the participants’ expectations, seem to be able to “socialize” the very phenomenological content of the hallucinatory experience.

If this suggests that bayesian approach of perception and models of social cognition of Bayesian inspiration (Carhart-Harris et al., 2014; Corlett et al., 2009) - such as the “Cultural Affordances” or the TTOM (“Thinking Throug Other Minds”) ones (Ramstead et al., 2016; Veissière et al., 2020) – may offer relevant tools for a better understanding of the socialization of hallucinations, some questions remain: What Bayesian models are most relevant to understanding the socialization of hallucinations? How do differences in interactional and communicative features during hallucination episodes in ritual contexts shape differences in the phenomenology of the psychedelic experience? What is the importance of the individual personality differences, and other individual perceptual styles (pathological or not) with an ‘organic’ basis in this dynamic? These questions should be explored through further studies.

### Hallucinogenic substances as tools for cultural transmission

Because of their ability to be deeply influenced by cultural context and social interactions, hallucinations appear to be very singular perceptions. The cognitive penetrability thesis of perception (which asserts that the content of perceptual experience can be influenced by prior or concomitant factors, such as beliefs, fears, and desires) is a controversial empirical hypothesis with regard to regular perceptions (Zeimbekis & Raftopoulos, 2015). Following my ethnographic data, however, it seems that this thesis can be asserted with respect to hallucinations.

We may then better understand the place occupied by hallucinogens in the social life of numerous indigenous groups of the Americas. As we have seen, the fact that the phenomenological content of hallucinations is partly shaped by cultural content supports the “experiential verification” (Dupuis, 2016) of cultural propositions. Regardless of culture, perceptual experience is indeed for human beings a fundamental source of epistemic justification—justification for believing that a proposition is true.

In addition to producing perceptions whose phenomenological content is strongly influenced by culture, psychedelic substances also have another remarkable property. Many observers have noted the ability of psychedelics to induce noetic feeling attributing strong self-relevance to the experience (Pahnke & Richards, 1970). The psychedelic experience is frequently marked by the striking subjective feeling of gaining unmediated knowledge. Information is usually received and believed as a revelation, i.e., without a subjective need for external validation or evidence (Timmermann et al., 2020).

Insofar as psychedelics are able to produce perceptions whose phenomenological content is strongly influenced by culture, their noetic property may enhance the significance and attribution of the reality of cultural worldviews as metaphysical, ontological, or supernatural claims. I claim that these two properties make hallucinogenic substances powerful potential vectors of cultural transmission. By producing a community of experience, hallucinogenic rituals are thus a vector of affiliation to the social group and are particularly efficient for the transmission of metaphysical propositions relating to the supernatural realm.

These observations contrast with models that have highlighted the central role of opacity in religious transmission (Atran, 2005; Sperber, 1996). These authors have pointed out that the understanding of ritual actions and the content of religious proposals are often opaque. In these contexts, the faithful “believe” in these propositions, i.e., do not understand them and have no first-hand evidence to justify them, but nevertheless accept them out of deference, because they are received from instituted authorities. These observations have shed light on the logic of transmission peculiar to certain religious traditions, such as Catholicism. However, this approach underestimates the embodiment of cultural knowledge (Csordas & Harwood, 1994; Luhrmann, 2012) which allows, for instance, concrete and tangible encounters with culturally postulated supernatural beings. Many traditions rely on these dynamics, such as shamanic initiation involving the use of hallucinogens (Déléage, 2009; Kopenawa, 2013), or numerous contemporary forms of Western religiosity, such as New Age or Evangelism (Houseman, 2012; Luhrmann, 2012).

In these contexts, the underpinnings of the transmission of cultural knowledge about the supernatural realm consist less in the transmission of propositional contents (“representations”), as it has been thought in the framework of cultural epidemiology (Sperber, 1996), than in a training inducing a transformation of the novice’s relationship to their perceptions and mental states. Religious transmission is then based less on the narrative and cognitive properties (i.e., minimally counter-intuitive category violations with high memorability) of these propositions (Boyer, 2001) than on this training of expectations, attention, and perception, which leads the novice to experience the content of these propositions in an embodied fashion.

To a certain extent, so-called “cognitive” anthropology has, in recent decades, diffused an excessively “representational” conception of religious transmission, which has been reduced to a set of automatic mental and propositional operations. The dynamics of the socialization of hallucinations draw our attention to the aspects neglected by this approach: the social learning processes, their ecological factors (Severi, 2004), and their experiential underpinnings.

## Conclusion

Like many ceremonial practices that mobilize the use of psychedelic substances, the hallucinogenic experience in Takiwasi is the object of a narrative elaboration which inscribes it in the “language game” of the social group. We argued here that these narrative reconstructions and the social interactions that frame the hallucinogenic experience, by educating the participant’s attention and affecting the categorization procedures of perceptions, further structure the way of organizing and interpreting the hallucinations. The symbolic knowledge acquired by the participants, the iconographic elements surrounding the visionary experience, as well as verbal and ritual interactions appear to be the main operators of a “socialization of hallucinations.” This dynamic finally seems to be able, by shaping the participants’ attention, emotions, and expectations, to formalize the very content of the visual and auditory imageries. By producing a community of experience, hallucinogenic rituals are thus powerful vectors of cultural transmission and affiliation to the social group.

I have attempted to shed light on what has been noted for almost a century by anthropologists: hallucinogenic experience is highly dependent on non-pharmacological factors such as expectation, preparation, and intention (set), as well as physical and social environments (setting) (Hartogsohn, 2016, 2017). These observations highlight the state of high suggestibility in which hallucinogenic substances place users (Carhart-Harris et al., 2015; Leary, 1961). If this is probably one of the underpinnings of the therapeutic efficacy of psychedelics, it also raises serious ethical questions in the current context of globalization of the use of these substances. Numerous hallucinogenic substances subject to these new uses, such as ayahuasca, have been recently banned in various countries of the global North. In France, the government has for instance expressed serious concern about the possible use of ayahuasca by so-called “cult” groups for the purpose of psychological manipulation and “brainwashing” (Miviludes, 2005).

This point raises the ethical implications of one of the striking features of psychedelic-induced experiences: their ability to increase truth to cultural propositions and reverence to the holders of these propositions. These features of the psychedelic experiences may act as a “double-edged sword” (Timmermann et al., 2020). While these mechanisms may drive therapeutic benefits, the ability of psychedelics to induce feelings of reverence and revelation might lead to problematic effects in certain contexts. These issues and concerns may consequently require further study.

Even if contemporary psychedelic research shows a growing interest in extra-pharmacological factors (Carhart-Harris et al., 2018), we are still lacking systematic studies on the underpinnings of these phenomena. A better understanding of the contextual mechanisms influencing the hallucinogenic experience could contribute to reducing drug harm by identifying and avoiding the risks specific to these substances and increasing potential positive effects (Hartogsohn, 2017). This appears to be a major and growing issue, as a renewed interest in these substances is generally observed in the Global North through the rise of shamanic tourism and the renaissance of psychedelic science.

In order to identify the extrapharmacological variables of the psychedelic experience, we should, as we suggested above, focus more closely on the ecology surrounding the use of hallucinogens at the level of the interactional setting. By providing thick descriptions of intricate contextual and interactional factors shaping the hallucinogenic experience, ethnographic studies can offer valuable insights. However, the understanding of the interactions between physiological conditions and social practices that are particularly readable in the psychedelic experience underlines the need for interdisciplinary methodologies articulating an anthropological approach to other disciplines such as phenomenology (Petitmengin et al., 2017) and the neurosciences (Studerus et al., 2012).

## Notes

1 The term “ayahuasca” refers to both a vine (*Banisteriopsis caapi*) and the beverage of which it is the main ingredient. This psychotropic and emetic brew is traditionally used in the Western Amazon as part of indigenous and métis shamanic practices and has aroused growing interest among the international public in recent decades as part of the emergence of shamanic tourism.

2 The term *curanderismo* refers to the practices of the métis and urban healers of the Peruvian Amazon (*curanderos*), and covers multiple specializations: medicinal plants, perfumes, prayer, midwife, etc. (Beyer, 2011).

3 *Malocas* are traditional Amazonian constructions, oval in shape with a conical roof.

4 Barks of a perfumed wood (*Bulnesia sarmientoi*) frequently used in the practices of the Peruvian *curanderismo*. Its use in Takiwasi is to purify and protect the ritual space.

5 Brown tobacco of a local variety (*Nicotiana rustica*).

6 *Agua florida* is a cologne used in the practices of Métis shamanism in South America as well as in Central America and the Caribbean as a purification, care, and protection tool.
